# Traumatic Cardiac Arrest: Scoping Review of Utilization of Resuscitative Endovascular Balloon Occlusion of the Aorta

**DOI:** 10.3389/fmed.2022.888225

**Published:** 2022-06-16

**Authors:** Makoto Aoki, Toshikazu Abe

**Affiliations:** ^1^Advanced Medical Emergency Department and Critical Care Center, Japan Red Cross Maebashi Hospital, Maebashi, Japan; ^2^Department of Emergency and Critical Care Medicine, Tsukuba Memorial Hospital, Tsukuba, Japan; ^3^Department of Health Services Research, University of Tsukuba, Tsukuba, Japan

**Keywords:** traumatic cardiac arrest, Resuscitative Endovascular Balloon Occlusion of the Aorta, return of spontaneous circulation (ROSC), mortality, review

## Abstract

Resuscitative Endovascular Balloon Occlusion of the Aorta (REBOA) is increasingly used in trauma resuscitation for patients with life-threatening hemorrhage below the diaphragm and may also be used for patients with traumatic cardiac arrest (TCA). Resuscitative thoracotomy with aortic cross clamping (RT-ACC) maneuver was traditionally performed for patients with TCA due to hemorrhagic shock; however, REBOA has been substituted for RT-ACC in selected TCA cases. During cardiopulmonary resuscitation (CPR) in TCA, REBOA increases cerebral and coronary perfusion, and temporary bleeding control. Both animal and clinical studies have reported the efficacy of REBOA for TCA, and a recent observational study suggested that REBOA may contribute to the return of spontaneous circulation after TCA. Although multiple questions remain unanswered, REBOA has been applied to trauma fields as a novel technology.

## Introduction

The mortality of traumatic cardiac arrest (TCA) remains high and was estimated to be 97.6% by a recent systematic review (1). The main cause of TCA is hemorrhagic shock (2); severe hemorrhage leads to decreased circulatory volume and the systemic pressure during chest compressions may be inadequate to achieve return of spontaneous circulation (ROSC).

Resuscitative Endovascular Balloon Occlusion of the Aorta (REBOA) is a resuscitative measure for the augmentation of cardiac and cerebral perfusion by controlling blood flow in the proximal aorta and hemorrhage from the distal portion. REBOA was first used more than 50 years ago (3); REBOA has been used for the treatments of ruptured abdominal aneurysm (4), postpartum hemorrhage (5), and trauma (6). Brenner et al. (6) first reported the use of REBOA for blunt and penetrating injuries associated with end-stage shock. Since then, REBOA became one of the modern technologies in trauma fields (7) and an increasing number of studies have been conducted on REBOA.

This article reviewed the current and future use of REBOA during TCA, including animal and human data. Literature was searched using PubMed database published between 1900 and 2020. The key words used for the search were combinations of “aortic balloon occlusion,” “intra-aortic balloon occlusion (IABO),” “REBOA,” and “traumatic cardiac arrest.” Though utilization of REBOA for non-traumatic cardiac arrest (NTCA) has been also spotlight and debated, the major difference between TCA and NTCA exists especially in pathophysiology, and we did not discuss the use of REBOA for NTCA in this scoping review.

### Indications of REBOA in TCA

#### Anatomical Aspect

**Figure 1:**
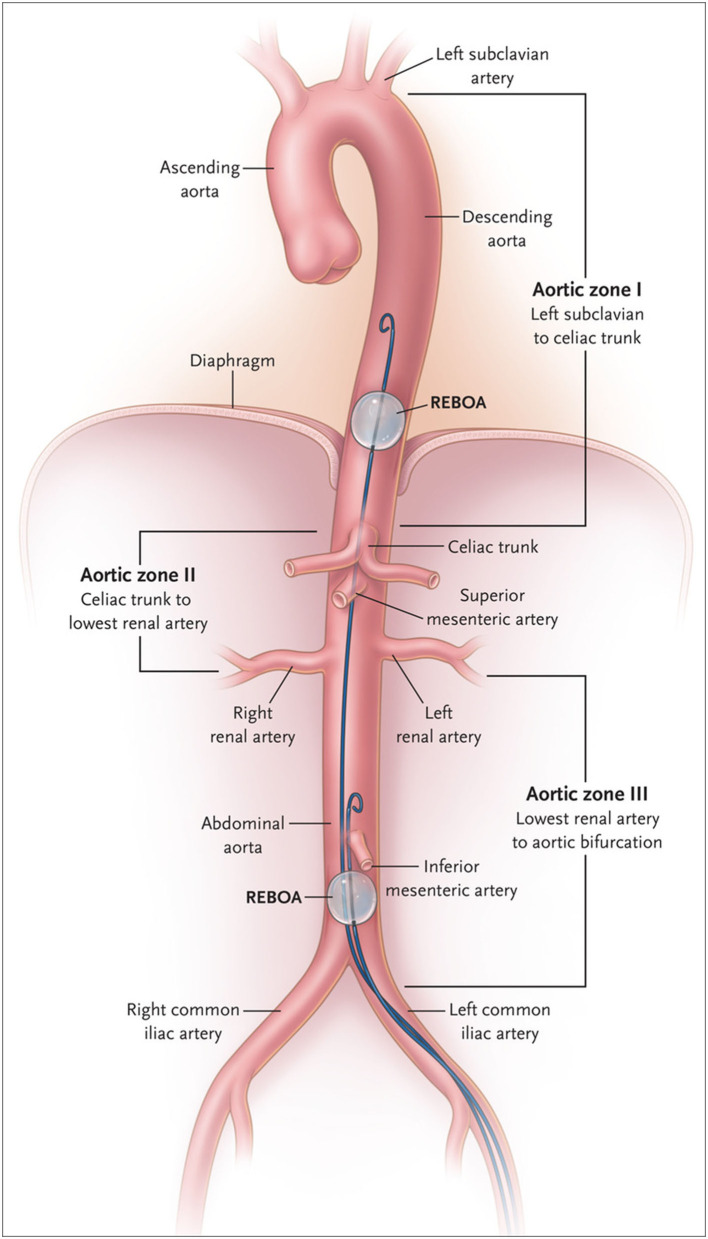
Classification of aortic zone using Resuscitative Endovascular Balloon Occlusion of the Aorta (REBOA). In Zone I, safe positioning of the balloon for control of infradiaphragmatic hemorrhage is shown; in Zone III, positioning for control of massive pelvic hemorrhage in the absence of a simultaneous abdominal source of hemorrhage is shown. From King (11). Copyright © 2022 Massachusetts Medical Society. Reprinted with permission from Massachusetts Medical Society.

The indication of use of REBOA in TCA should be discussed based on the anatomical and physiological aspects. REBOA is generally indicated for use in patients with bleeding below the diaphragm. The use of REBOA for patients with major hemorrhage above the diaphragm, such as traumatic brain injury (8) or thoracic injury (9), could increase hemorrhage. The joint statement from American College of Surgeons Committee on Trauma stated that REBOA is contraindicated in the setting of major thoracic hemorrhage or pericardial tamponade (10). REBOA is placed in Zone 1 or 3 (Figure 1) (11). Zone 1 is the distal thoracic aorta, which is selected for the control of severe intra-abdominal or retroperitoneal hemorrhage, or in patients with traumatic arrest (12). Zone 3 is the distal abdominal aorta, which is selected for patients with severe pelvic, junctional, or proximal lower extremity hemorrhage (11, 12).

### Physiological Changes After REBOA Deployment After TCA

**Figure 2:**
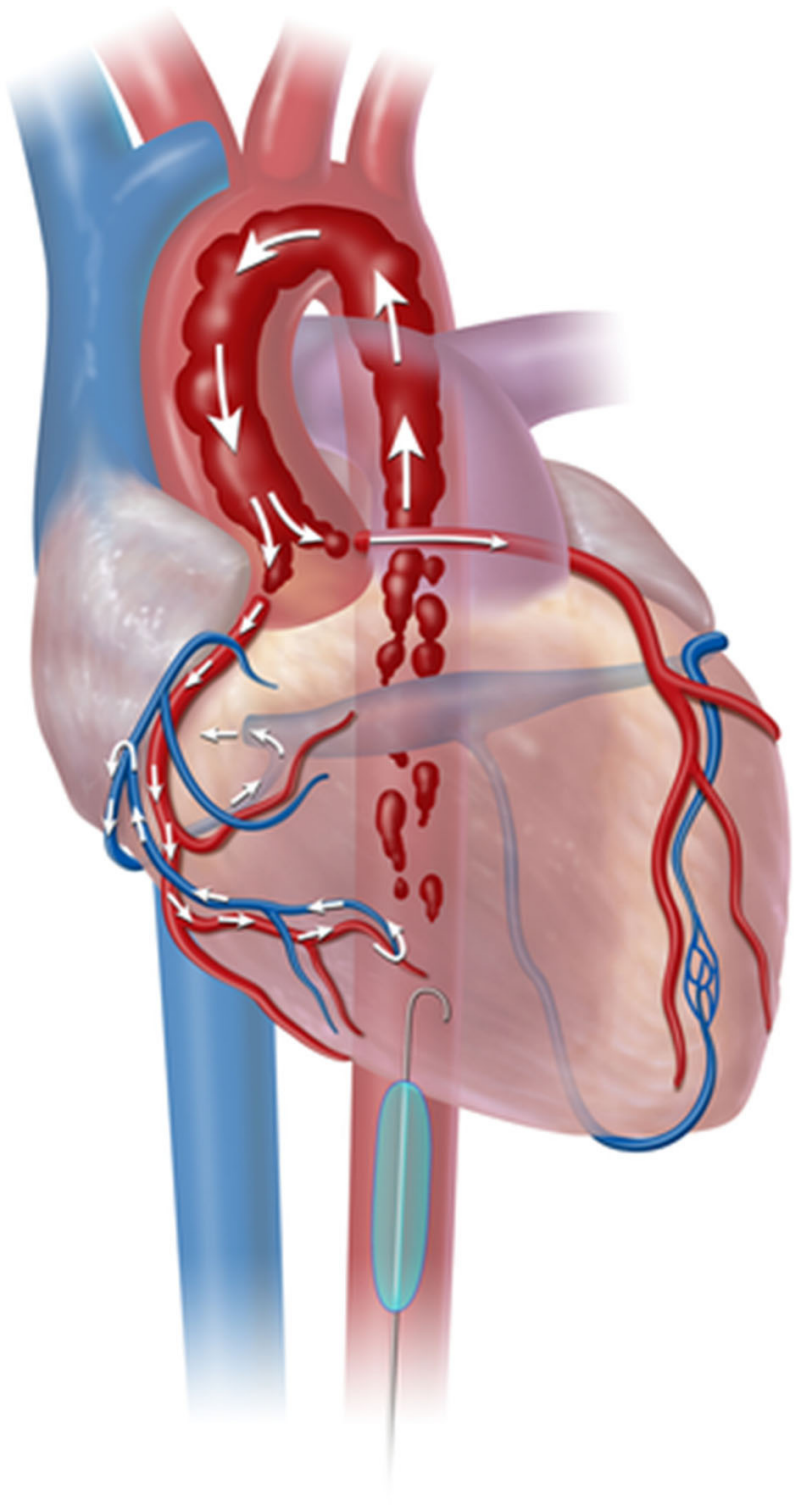
Resuscitative Endovascular Balloon Occlusion of the Aorta (REBOA) deployment in aorta Zone 1. Zone 1 aortic occlusion with REBOA allows the cardiac output generated from cardiopulmonary resuscitation to be directed toward cardiac and cerebral vessels. From Nowadly et al. (14). Copyright © 2020 Reprinted with permission from J Am Coll Emerg Physicians Open.

Current expert consensus and clinical guidelines state that trauma patients with an initial systolic blood pressure <90 mmHg who do not respond at initial fluid or blood product administration are potential candidates for REBOA use (12, 13). However, REBOA is modestly indicated for TCA patients, albeit with limited evidence. Current guidelines state that REBOA is indicated for patients arriving in arrest from injury due to presumed life-threatening hemorrhage below the diaphragm; in these patients, REBOA should be used within the same time period as resuscitative thoracotomy-aortic cross clamping (RT-ACC) (12). The physiological indication of REBOA for TCA includes patients with signs of life on arrival, which is comparable to the indications of RT-ACC. Physiologically, aortic occlusion (AO) during hemorrhagic shock including TCA results in increases in coronary blood flow (Figure 2) (14), cardiac output, mean arterial pressure, carotid blood flow, and partial oxygen pressure of the brain (15, 16). AO simultaneously minimize the major hemorrhage below the diagram maintaining proximal aortic pressure, and contributing to resuscitation and surgical repair of hemorrhage (17).

### Superiority of REBOA to RT-ACC for TCA

RT-ACC is maximally invasive procedure and produces additional severe thoracic injury (17), on the other hands, REBOA is less invasive. Another feature of REBOA is that we could control of distal organ perfusion by adjusting balloon volume. If TCA patient was resuscitated by initial resuscitation, the hemodynamics may be controlled using the inflation balloon volume. If the resuscitated patients could maintain acceptable hypotension (permissive hypotension), partial REBOA could maintain the distal organ perfusion and prevent the ischemic complications (18). Besides, adjusting balloon volume enabled to temporarily control the bleeding, carry out surgical treatment in a bloodless field and identify the site of bleeding (19). AO by RT-ACC cannot be unlocked unless hemostatic treatment performed, and the distal organ perfusion was not maintained.

Notably, REBOA does not interrupt closed chest-compressions, which is a significant advantage for TCA patients (20). A prospective observational study of 22 REBOA cases and 28 RT cases analyzed the interruptions in the chest compressions and reported fewer interruptions in patients who had received REBOA compared to RT. Compression was continued 86.5% of the time for REBOA and 35.7% of the time for RT (20). R Adams Cowley Shock Trauma Center confirmed that the end-tidal carbon dioxide value after aortic occlusion was higher in REBOA compared to RT-ACC, and the rate of ROSC was higher in REBOA compared to RT-ACC [20/33 (60.1%) vs. 5/18 (33.3%), respectively; *p* = 0.04] (21). Conversely, the disadvantage of REBOA is that it may take longer to perform AO by REBOA compared to RT-ACC. The team at R Adams Cowley Shock Trauma Center reported that the time to AO was shorter for RT-ACC compared to REBOA [median time to AO was 317.5 (IQR 227–551) s for RT-ACC vs. 474 (IQR 431–572) s for REBOA] (22). However, REBOA had shorter time to AO once arterial access was established [median time to AO was 245 (179–295.5) s once common femoral artery (CFA) access was established] (22). In addition, REBOA with a wire-free device was commercialized in the USA to achieve earlier time to AO (23). REBOA with a wire-free device could be directly inserted into the aorta without guidewire and this device can be inserted by one provider, and shorten the time to AO (23). Conventional REBOA is inserted by over the wire technique and long stiff guidewire is needed.

Additionally, we described the superiority of RT-ACC compared to REBOA. First, TCA patients with thoracic injury should be resuscitated by RT-ACC, which could immediately control major hemorrhage from the thoracic regions and the shock from cardiac tamponade. Therefore, multiply injured patients with thoracic injury tended to be selected by RT-ACC (24). Second, CFA access is generally difficult among TCA patients compared to hypotensive patients (22, 25). Besides, even if CFA access was achieved, REBOA sometimes may not be deployed for patients with severely tortuous aorta (25). Therefore, it is preferable to select RT-ACC for TCA patients with difficulty in CFA access or severe aortic tortuosity.

Practically, the conversion from RT-ACC to REBOA was reported and previous report showed 30 cases among 106 REBOA cases were RT and REBOA combined cases (26). After TCA patients underwent RT-ACC and achieved ROSC, the patient would suffer from loss of body heat that was potentially caused by exposed pleural cavity and oozing from the incision site of chest. Then, closing the chest wall after RT and converting from RT to REBOA could be a practical choice.

### Clinical Research of REBOA for TCA

Most clinical research regarding REBOA in trauma fields excluded TCA patients and limited evidence exists for the utilization of REBOA in TCA (Table 1). The mortality of REBOA patients significantly varies with the presence or absence of vital signs necessitating CPR (28). Therefore, previous investigators excluded the TCA patients. An observational prospective study from the American Association for the Surgery of Trauma (AAST) Aortic Occlusion (AO) for Resuscitation in Trauma compared REBOA and RT-ACC for trauma patients requiring AO, including those with TCA (27). In this cohort, 34.7% of REBOA patients (16/46) underwent CPR during initial AO by REBOA and the mortality of REBOA patients who underwent CPR was unknown. In this study, the mean time from initiation of procedure to successful AO did not vary between REBOA and RT-ACC (6.6 vs. 7.2 min, respectively; *p* = 0.842) (30); therefore, the clinical use of REBOA for TCA may be feasible as an alternative to RT-ACC (30). Subsequent reports from the American Association for the Surgery of Trauma (AAST) Aortic Occlusion (AO) (Aorta-2) for Resuscitation in Trauma showed no statistical difference in terms of mortality among TCA patients between REBOA and RT-ACC (96.4 vs. 97.7%, respectively) (27). A trauma registry from Japan (Japan Trauma Data Bank) reported a possible survival benefit of REBOA for TCA compared to RT-ACC (29). The major difference between Aorta-2 and JTDB was whether the time of initiation of CPR was known or not. JTDB did not report whether REBOA was inserted before or after CPR (29). A single center study from the R Adams Cowley Shock Trauma Center reported comparable mortality of 90.0% and ROSC of 58.0% (29/50) among TCA patients (31). A recent prospective observational study at US 6 Level-1 trauma centers reported that 59% achieved ROSC among TCA patients (32). Taken together, the conclusion was that REBOA in TCA patients due to non-compressible torso hemorrhage below the diagram is preferable (32).

**Table 1:** Summary of previous studies of mortality of REBOA for TCA.

**References**	**Type of study**	**Place of study**	**Duration**	**Patient indication**	**Outcomes of REBOA patients (%)**
Moore et al. (18)	Dual-center retrospective	United States	Jan 2012–Jun 2013	REBOA vs. RT-ACC	In-hospital mortality: 7/7 (100) Mortality in ED: 4/7 (57.1%)
Dubose et al. (27)	Prospective observational, multicenter	United States	Nov 2013–Feb 2015	REBOA vs. RT-ACC	N.A
Brenner et al. (28)	Prospective observational, multicenter	United States	Nov 2013–Jan 2017	REBOA vs RT-ACC	In-hospital mortality: 54/56 (96.4%) Mortality in ED: 29/56 (51.8%)
Brenner et al. (29)	Retrospective observational, single-center	United States	Feb 2013–Jan 2017	REBOA	In-hospital mortality: 45/50 (90.0%) Morality in ED: 39/50 (78.0%) ROSC: 29/50 (58.0%)
Yamamoto et al. (30)	Retrospective cohort, multicenter	Japan	Jan 2004–Mar 2019	REBOA vs. RT-ACC	In-hospital mortality: 139/144 (96.5%)
Moore et al. (31)	Prospective observational multicenter	United States	May 2017–Jun 2018	REBOA	In-hospital mortality: 16/17 (94.1%) Mortality in ED: 7/10 ROSC: 10/17 (58.8%)

*REBOA, resuscitative endovascular balloon occlusion of the aorta; TCA, traumatic cardiac arrest; RT, resuscitative thoracotomy; ACC, aortic cross-clamping; NA, not applicable*.

### Unresolved Problems of REBOA for TCA

A joint statement from the American College of Surgeons Committee on Trauma (ACS COT) and the American College of Emergency Physicians suggest a longest occlusion time of <15 min for Zone 1 (12). TCA patients already exposed to ischemia were more prone to ischemia-reperfusion injury; therefore, it is unclear how long the TCA patient can accept the Zone 1 inflation. Expert opinion recommends deflating the balloon if the TCA patient tolerates the deflation by proximal aortic pressure. Full occlusion can be switched to partial occlusion once TCA patients achieve ROSC; however, the switch from full to partial occlusion is practically difficult until definitive hemostatic treatment is completed (33).

Another problem to consider is REBOA-related complications (28, 34). Although REBOA is less invasive, major complications may occur. Recent review summarized the complications with associated REBOA, and noted complications can arise in arterial access (i.e., vessel injuries, embolization, air emboli, and peripheral ischemia), balloon inflation (i.e., rupture of the balloon and aortic injury), during occlusion (i.e., other arterial injury, retroperitoneal hemorrhage, lactic acidosis, organ dysfunction, and limb ischemia), deflation (i.e., ischemic reperfusion injury), and removal of the sheath (i.e., distal thrombus and arterial dissection) (34). A nationwide database study (American College of Surgeons Trauma Quality Improvement Program data set) reported high complication rates such as acute kidney injury and lower leg amputations (35) and we had to know REBOA may cause serious complications. REBOA has been a more advanced and lower profile device (36) and complication rates were expected to be lower (36); however, several complications still exist (32).

## Conclusions

REBOA is one of the modern technologies among the trauma field, which has led to a paradigm shift. Recent clinical evidence suggests that the efficacy of REBOA was comparable to RT-ACC for TCA patients; in addition, REBOA may contribute to achieving ROSC and additional definitive hemostatic treatment. However, the mortality of TCA patients remains high and further prospective studies are warranted to validate the efficacy of REBOA for TCA patients.

## Author Contributions

MA conceived the idea for this scoping review and drafted the manuscript. TA revised the manuscript. Both authors critically reviewed and approved the final manuscript.

## Funding

This study was supported in part by research grants from The General Insurance Association of Japan, 21-1-100.

## Conflict of Interest

The authors declare that the research was conducted in the absence of any commercial or financial relationships that could be construed as a potential conflict of interest.

## Publisher's Note

All claims expressed in this article are solely those of the authors and do not necessarily represent those of their affiliated organizations, or those of the publisher, the editors and the reviewers. Any product that may be evaluated in this article, or claim that may be made by its manufacturer, is not guaranteed or endorsed by the publisher.
